# Mechanochemistry-assisted synthesis of hierarchical porous carbons applied as supercapacitors

**DOI:** 10.3762/bjoc.13.130

**Published:** 2017-07-06

**Authors:** Desirée Leistenschneider, Nicolas Jäckel, Felix Hippauf, Volker Presser, Lars Borchardt

**Affiliations:** 1 Institute of Inorganic Chemistry, Technische Universität Dresden, Dresden, Germany; 2 INM - Leibniz Institute for New Materials & Saarland University, Saarbrücken, Germany; 3 Department of Materials Science and Engineering, Saarland University, Saarbrücken, Germany; 4 Fraunhofer Institute for Material and Beam Technology IWS, Dresden, Germany

**Keywords:** electrochemical energy storage, mesoporous, microporous, solvent-free, supercapacitor, templated carbon

## Abstract

A solvent-free synthesis of hierarchical porous carbons is conducted by a facile and fast mechanochemical reaction in a ball mill. By means of a mechanochemical ball-milling approach, we obtained titanium(IV) citrate-based polymers, which have been processed via high temperature chlorine treatment to hierarchical porous carbons with a high specific surface area of up to 1814 m^2^ g^−1^ and well-defined pore structures. The carbons are applied as electrode materials in electric double-layer capacitors showing high specific capacitances with 98 F g^−1^ in organic and 138 F g^−1^ in an ionic liquid electrolyte as well as good rate capabilities, maintaining 87% of the initial capacitance with 1 M TEA-BF_4_ in acetonitrile (ACN) and 81% at 10 A g^−1^ in EMIM-BF_4_.

## Introduction

Porous carbons are key components in many energy and environmentally-relevant applications, such as catalysis [1], gas storage and separation [2–3], and electrochemical energy storage [4–6]. Among them, activated carbons derived from natural precursors such as coconut shells are widely used in industrial applications [7]. Due to their high specific surface area, predominantly provided by micropores, they can physisorb large quantities of molecules. They are also particularly suitable as electrode materials for supercapacitors, in which the energy storage is based on the electrosorption of electrolyte ions on the electrode surface [8–10]. These micropores are usually introduced by physical or chemical activation, often leading to broad pore-size distributions and non-uniform pore structures [11]. However, for size-selective applications [12], non-uniform broad pore-size distributions lead to lower performance metrics [13–14]; they are also detrimental to derive clear statements about structure–performance relationships for fundamental research, such as the influence of the pore size and the pore structure on (electro)sorption in energy storage devices [15–17]. Moreover, purely microporous carbons suffer from diffusion limitations resulting in low electrochemical performances at high charge/discharge rates [4,18–19]. Larger pores, like mesopores, or hierarchical micro-meso-macroporous pore systems, facilitate fast ion transport through the carbon pore network [20–21]. Therefore, synthesis approaches leading to such pore systems are highly desirable to improve the electrochemical performance of carbon supercapacitors.

A well-established strategy for designing the porosity of carbon materials involves hard or soft templates [22–24]. Hard-templating utilizes metal oxide nanoparticles [25] and salts [26–28], which have to be synthesized in advance. Soft-templating employs surfactants or other structure-directing molecules, which self-assemble to form the desired template [29–30]. A severe disadvantage of both routes is the need of large amounts of solvents, eventually accumulating as waste during the process. Moreover, these approaches require multiple synthesis steps, including template synthesis, calcination, impregnation, pyrolysis, and template removal. Therefore, the preparation of porous carbons with a tailored pore structure by conventional templating processes is often time and cost-intensive and environmentally unfavorable. For a more sustainable carbon production, especially in industrial scale, it is necessary to reduce the number of synthesis steps and to minimize waste accumulation, at best by avoiding any solvents [7,31].

Lately, mechanochemistry has gained momentum in organic chemistry [32–34]. The initiation of chemical reactions by mechanical forces enables organic and inorganic syntheses without the use of any solvent within short reaction times of only few minutes [32,35]. A mechanochemical synthesis also enables high yields, making it a promising approach to obtain carbons and carbon precursors [36–37]. So far, mechanochemical reactions for the synthesis of porous carbon materials have rarely been used [38]. For example, the preparation of nanocarbon structures such as graphene sheets or fullerenes [39–41] as well as porous carbonaceous polymers [42–43] have been conducted mechanochemically. Our work demonstrates that a templating approach can be transferred into the solvent-free environment of a ball mill, and thus simplify the synthesis of hierarchical porous carbons drastically. Moreover, it is the first proof that even well-defined carbon pore structures can be derived making use of solid-state conditions like ball-milling. In detail, we apply the Pechini method, an approach commonly used for the synthesis of uniform metal oxide nanoparticles to synthesize a titanium(IV) citrate-based polymer [44–45]. The Pechini method is applicable to synthesize templated mesoporous carbons [46–49], but has never been utilized for a solvent-free and rapid process based on mechanochemistry.

**Figure 1:**
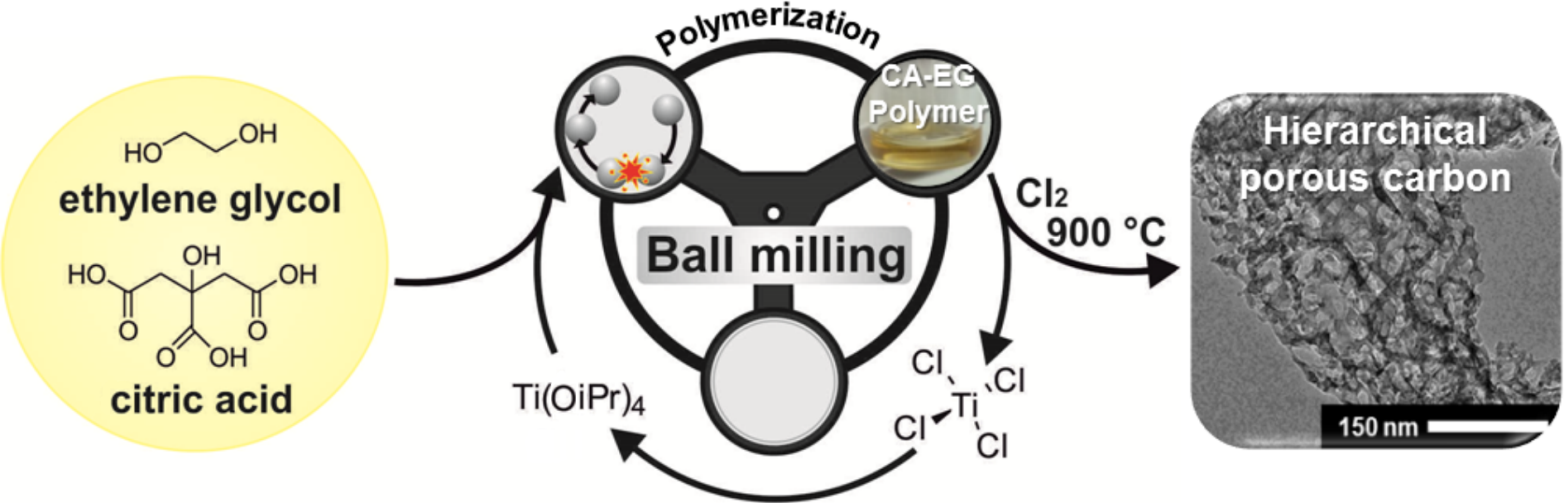
Synthesis of hierarchical porous carbons by mechanochemical polymerization of ethylene glycol (EG) with citric acid (CA) and Ti(IV) isopropoxide as a porogen, resulting in CA-EG polymers. After carbochlorination at 900 °C hierarchical carbons are obtained. The byproduct TiCl_4_ can be recycled to be used as Ti(IV) isopropoxide, and acts as porogen in further syntheses.

The combination of this approach with a high temperature chlorine treatment enabled us to simultaneously carbonize the polymer and selectively remove the titania. By this way, we obtained a hierarchical carbon with a high pore volume, high specific surface area, tunable mesopore volume, and a well-defined pore-size distribution. The material was further investigated as supercapacitor electrode using organic and ionic liquid electrolytes (Figure 1).

## Results and Discussion

### Mechanochemical synthesis of the polymeric precursor

**Figure 2:**
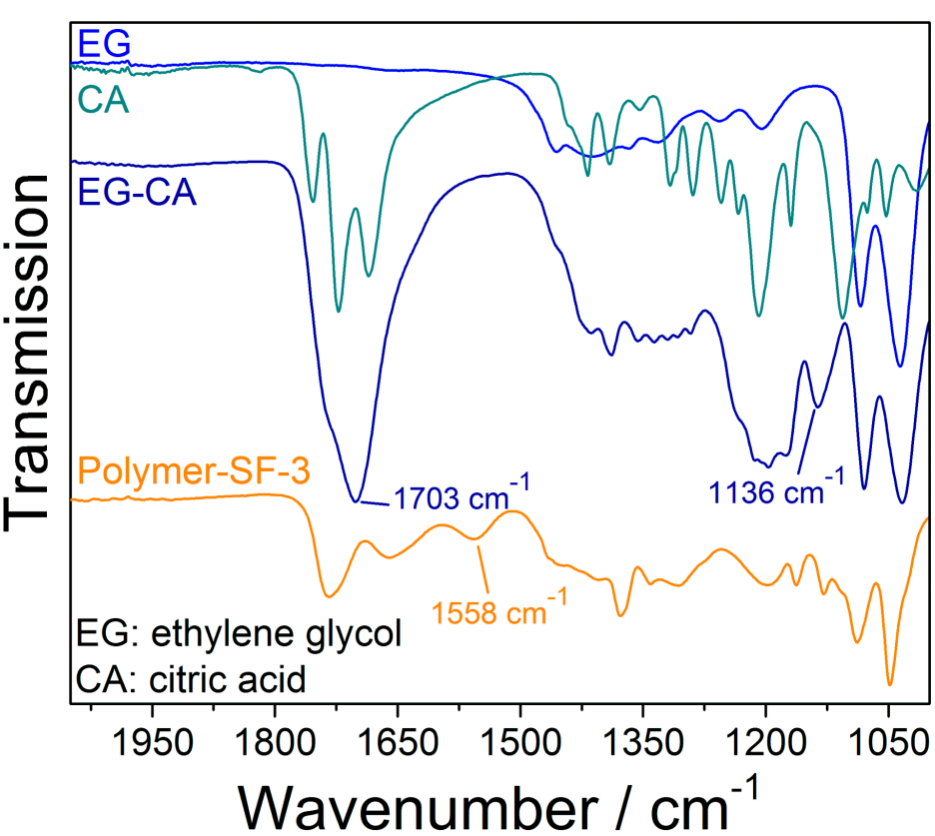
Infrared spectra of the monomers ethylene glycol (EG, blue) and citric acid (CA, green blue), the metal-free polymer achieved by 5 min ball milling with ZrO_2_ balls (*d* = 15 mm) (EG-CA, dark blue) and the polymeric precursor after adding titanium(IV) isopropoxide (Polymer-SF-3, orange).

For a typical synthesis, ethylene glycol (EG), citric acid (CA), and titanium isopropoxide (TIPP) were ground with a molar ratio of 3:1:1 in a ZrO_2_ milling cup for 5 min. A practical indicator for a successful mechanochemical reaction is a color change. The white and colorless educts turn to a yellow polymer with a honey-like texture. We first characterized the polymerization of the educts induced by mechanochemical forces by IR spectroscopy (Figure 2). Two bands at 1703 cm^−1^ and 1136 cm^−1^ appear, indicating the formation of the polyester (EG-CA). Likewise, the characteristic bands of the educts (CA: 1210 cm^−1^; EG: 1418 cm^−1^) become less pronounced and much broader as they are gradually consumed by the mechanically-induced polymerization. The spectrum of the Ti-containing polymer (Polymer-SF-3) displays the appearance of a band at 1558 cm^−1^, which corresponds to titanium, bidentate to a carboxylic group [50]. Additionally, the blue-shift of the vibration at 1703 cm^−1^ indicates complexation [51]. The sample Polymer-SF-3 was investigated by matrix-assisted laser desorption/ionization with a time-of-flight mass spectrometer (MALDI–TOF) revealing a weight-averaged molar mass (*M*_w_) of 2015.6 g mol^−1^, which is equivalent to 6 monomeric units.

### Synthesis of the hierarchical porous carbons

After the mechanochemical synthesis, we conducted a carbochlorination reaction, leading to the carbonization of the precursor and the removal of the dispersed titanium species (Equation 1). This process is comparable to the industrial Kroll process and responsible for the generation of mesopores that correspond to the size of the former titania nanostructures [25]. While titanium is removed as gaseous TiCl_4_, oxygen is extracted as CO, whereby carbon is being partially consumed as well. This partial carbon removal leads to an etching of the carbon framework and an in situ formation of micropores, surrounding the formed mesopores. Consequently, a hierarchical porous carbon material is formed [52]. The resulting byproduct TiCl_4_ is a valuable precursor for Ti-containing [53] chemicals like Ti(IV) isopropoxide, other Ti-alkoxides, or can directly be applied in the presented synthesis approach once again [52].

[1]
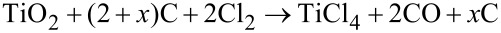


**Figure 3:**
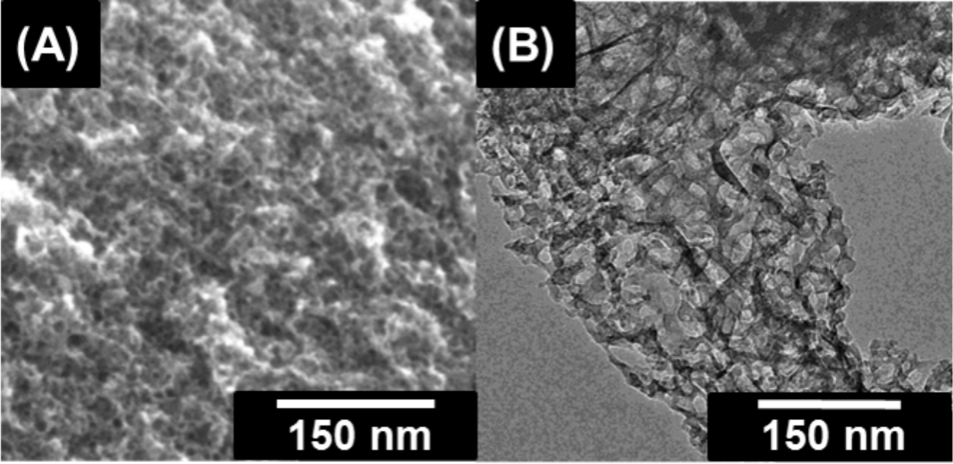
SEM (A) and TEM (B) images of the Carb-SF-3 sample.

**Figure 4:**
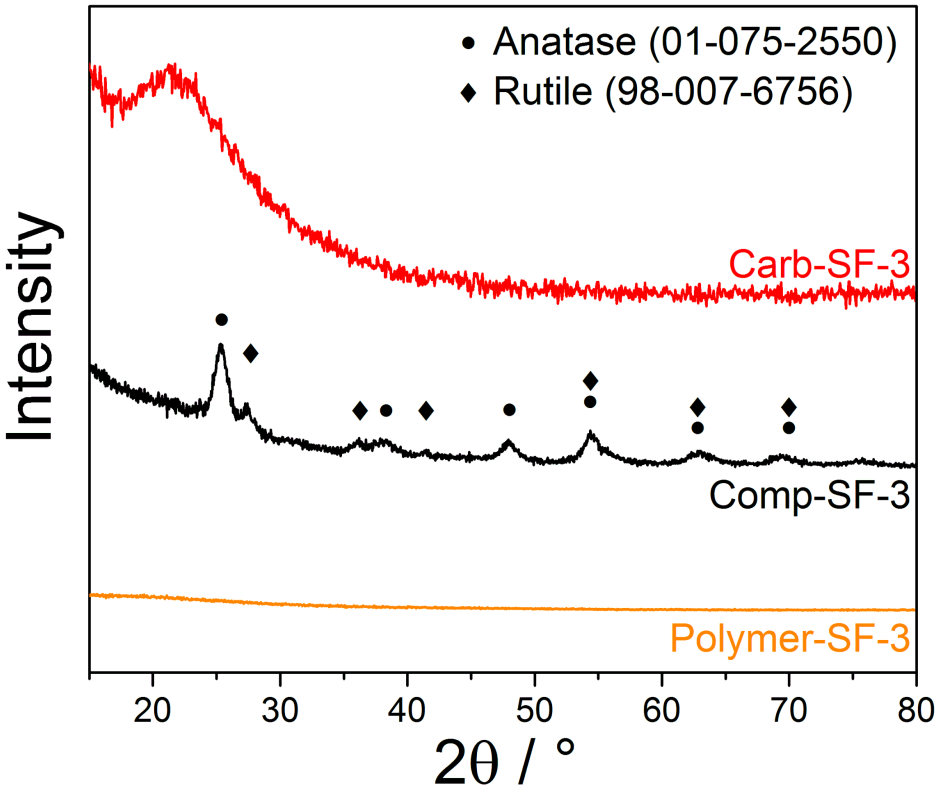
XRD-pattern of the polymeric precursor (Polymer-SF-3, orange), the carbonized composite (Comp-SF-3, black) and the carbon received by chlorine treatment (Carb-SF-3, red).

Scanning and transmission electron micrographs indicate that the carbon material exhibits spherically shaped mesopores (Figure 3), corresponding to the removal of TiO_2_ particles which have been formed during the pyrolysis (Figure 4). The pores are homogenously distributed, resulting in a well-connected pore system of the carbon material (Figure 3A,B).

To display the complete removal of the porogenous TiO_2_, we compared the XRD pattern (Figure 4) of the material at different synthesis steps: after mechanochemical polymerization (Polymer-SF-3), after temperature treatment but before Cl_2_ addition (Comp-SF-3), and after the carbochlorination reaction (Carb-SF-3).

The absence of X-ray reflections confirms the amorphous nature of the polymeric precursor. Titanium is atomically coordinated and distributed within the polymer and does not form crystalline TiO_2_ nanoparticle domains. After carbonization broadened reflections occur due to the conversion of the bidenated Ti atoms to TiO_2_ nanoparticles of the rutile and anatase modification. We calculated the average domain size of crystalline TiO_2_ from the reflections at 25.4°, 48.0°, and 54.5° 2θ to be 6–9 nm after background adjustment using the Scherrer equation. Carbochlorination will remove these nanoparticles, leading to mesopores of comparable size. The XRD pattern of the carbon shows the broad (002) reflection of nanocrystalline carbon, but all signals related to titania have disappeared. This assumption was further supported by EDX measurements (Table 1), showing a Ti content below the detection limit.

**Table 1:** Porosity and composition data summary for the different samples.

Samples^a^	SSA_BET_^b^ / m^2^ g^−1^	SSA_DFT,microc_ / m^2^ g^−1^	V_total_^d^ / cm^3^ g^−1^	V_meso_^e^ / cm^3^ g^−1^	V_micro_^f^ / cm^3^ g^−1^	d_mesopore_^g^ / nm	Ti content^h^ / %

Polymer-SF-3^i^	–	–	–	–	–	–	15.7^j^
Comp-SF-3	298	185	0.17	0.10	0.07	–	45.8 ± 13.4
Carb-SF-1	1442	623	1.34	1.11	0.23	4–14	<d.l.
Carb-SF-2	1532	480	1.62	1.43	0.19	4; 6–12	<d.l.
Carb-SF-3	1814	558	1.83	1.62	0.23	4; 6–14	<d.l.
Carb_HF_-SF-3	291	144	0.20	0.14	0.06	–	n.d.
Comp-LA-3	312	173	0.18	0.14	0.04	–	62.8 ± 16.7
Carb-LA-3	1590	445	1.59	1.41	0.18	4; 6–13	<d.l.
Carb_HF_-LA-3	706	123	0.62	0.48	0.14	4–12	11.1

^a^Sample code x−y−z as follows, x describes the material after polymerization (Polymer), after heat treatment (Comp) and after carbochlorination (Carbon), the indices HF notices that the template was removed by HF instead of Cl_2_, y describes the reaction conducted solvent-free (SF) or liquid-assisted (LA), z describes the ratio of EG to CA. ^b^Specific surface area (SSA) determined in a pressure range of 0.05 < *p*/*p*_0_ < 0.2. ^c^Specific surface area of the mesopores determined by QSDFT below 2 nm. ^d^Total pore volume determined at *p*/*p*_0_ = 0.99. ^e^Mesopore volume = *V*_total_ – *V*_micro_. ^f^Micropore volume determined by QSDFT below 2 nm. ^g^Mesopore size determined by QSDFT kernel for slit-shaped, cylindrical, spherical pores using the adsorption branch. ^h^Ti content determined by EDX measurement, <d.l. = below detection limit. ^i^The polymer is non-porous. ^j^The polymer is not stable in the electron beam; therefore, the composition must be determined from thermogravimetric analysis (TG) rather than from EDX.

**Figure 5:**
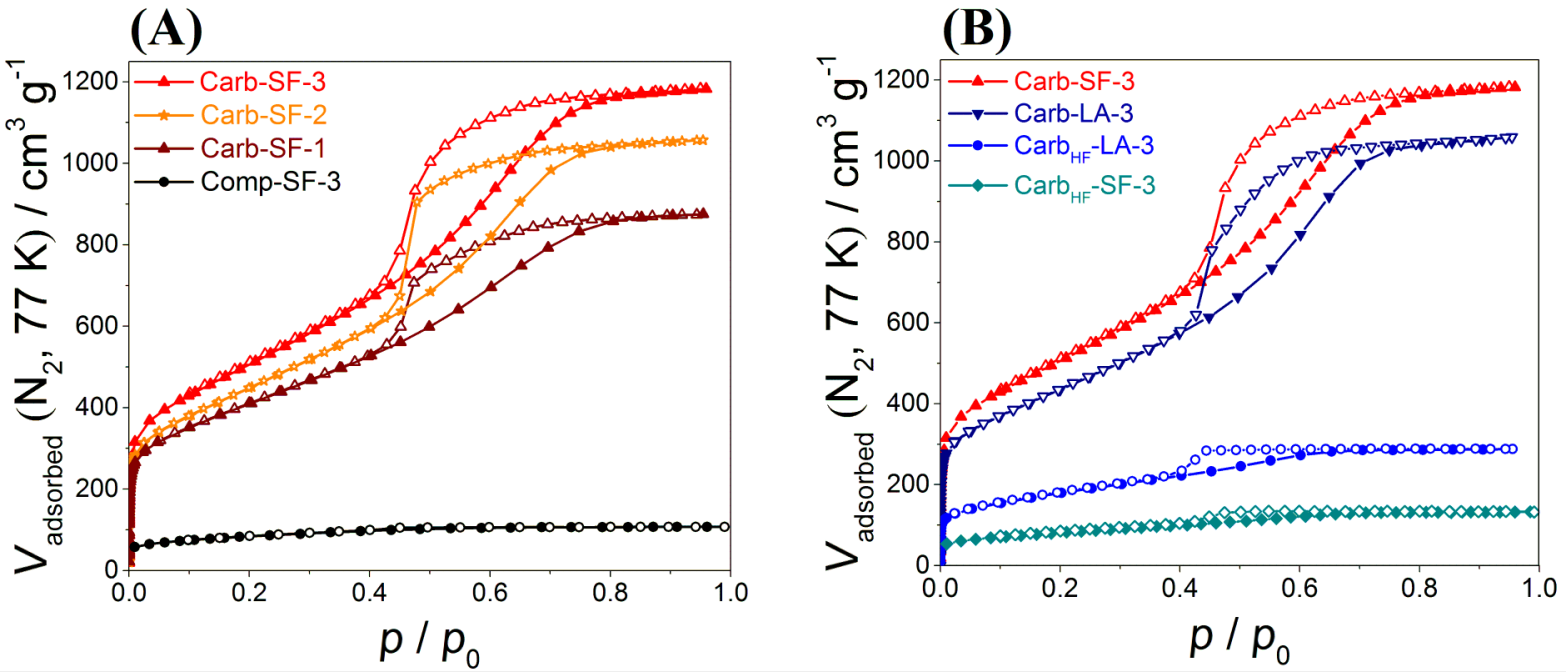
Nitrogen physisorption isotherms for carbon samples achieved from (A) different amounts of ethylene glycol and (B) different syntheses.

The pore structure of the materials was analyzed by nitrogen physisorption (Figure 5A). Neither the polymer (Polymer-SF-3) nor the carbonized composite material (Comp-SF-3) show a significant porosity (Table 1). This was expected since the porogens have not been removed during this step in the synthesis. The low specific surface area of 298 m^2^ g^−1^ for the composite arises from chemical activation processes of volatile functional groups such as carboxylic acids, which form micropores during pyrolysis.

**Figure 6:**
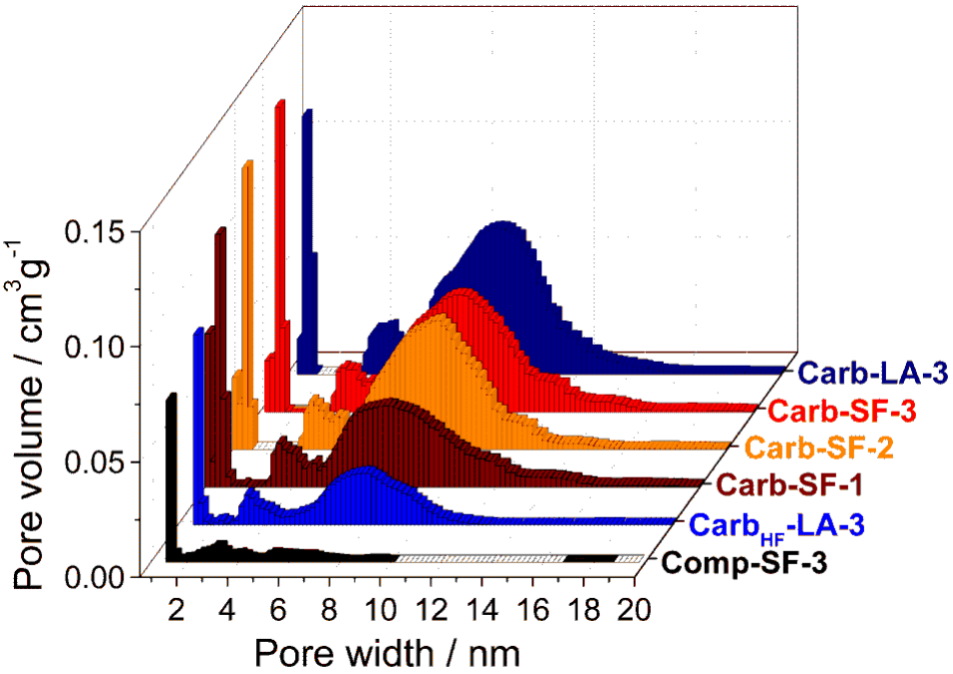
Volume histogram of the different samples calculated using a QSDFT-kernel for slit, cylindrical and spherical pores on the adsorption branch.

After carbochlorination at 900 °C, the obtained carbon (Carb-SF-3) shows a well-developed micro- and mesoporosity, obvious due to a type IV isotherm and a high nitrogen uptake at low relative pressure, which is attributed to the amount of micropores in the samples. The obtained material has a high specific surface area of up to 1814 m^2^ g^−1^ and a pore volume of 1.83 cm^3^ g^−1^. The contributions of the individual pore-size increments are shown in Table 1 and Figure 6. The carbons possess narrowly distributed micropores with an average size of 0.96 nm (due to the in situ activation process), as well as mesopores with an average diameter of 8 nm (due to the removal of TiO_2_ nanodomains) (Figure 6, Equation 1). The mesopore diameter (Table 1 and Figure 6) aligns very well with the calculated domain size of TiO_2_ nanocrystals derived from the Scherrer equation. A more precisely evaluation of the hierarchical pore structure is given in Table S1 in Supporting Information File 1.

We further investigated the influence of the EG to CA ratio on the porosity of the material, while keeping the TIPP to CA ratio constant (1:1). The pore volume increased with a higher content of ethylene glycol from 1.34 cm^3^ g^−1^ for a ratio of 1:1 to 1.83 cm^3^ g^−1^ for a ratio of 3:1. This is mainly attributed to the increased mesopore volume, while the micropore volume stayed nearly the same (0.20 cm^3^ g^−1^, Table 1). The higher mesopore content originates from the higher amount of ethylene glycol, which promotes the formation of more slightly larger pores. This process finally leads to a higher mesopore volume at the expense of a narrower pore-size distribution (Figure 6). The EG ratio does not impact the particle size of the TiO_2_ nanostructures and thus the pore-size distributions are similar for all investigated materials (average diameter of 8 nm). The reaction in total absence of EG yields white powder next to purely black carbon phases (see Figure S2, Supporting Information File 1). This indicates that the carbon content was insufficient and demonstrates the inevitable role of EG.

To further investigate the mechanochemical polymerization and the carbochlorination step, we conducted the synthesis under liquid-assisted conditions while adding ethanol as a solvent to see if there is a difference in the polymerization and investigated an alternative template removal approach based on etching with hydrofluoric acid (HF) as well [54]. The latter is a common process used in industry [55]. However, by doing so, it is impossible to remove the porogenous TiO_2_ completely from the carbon matrix (sample Carb_HF_-SF-3). The resulting material still contained 11.1 wt % of Ti and did not show a high porosity (Figure 5B and Table 1) with a pore volume of 0.20 cm^3^ g^−1^ and a surface area of 291 m^2^ g^−1^. Indeed, the high temperature chlorination reaction is essential to obtain the full porosity of the desired carbon. However, when we conducted the mechanochemical polymerization in the presence of small amounts of ethanol (liquid-assisted grinding, LA), the porogens could be partially removed by HF (Carb_HF_-LA-3). We assume that the carbon matrix obtained from a solvent-free approach is possibly denser and thus the diffusion of HF to the particles is inhibited (incomplete removal). This aligns with the assumption that the energy-input and accordingly the embedding of the particles are higher in case of solvent-free syntheses. When we compare SF and LA samples (both received by carbochlorination), we observe full template removal and obtain hierarchical porous carbons with high surface area and pore volume (Table 1 and Figure 5B). The addition of a solvent during the mechanochemical synthesis does not influence the porosity of the composite materials, since both composites (Comp-SF-3 and Comp-LA-3) provide the same pore volume (0.2 cm^3^ g^−1^) and SSA (300 m^2^ g^−1^, Table 1). However, the carbons derived after carbochlorination differ in their porosities. The solvent-free synthesis results in a higher specific surface area and pore volume as compared to the liquid-assisted approach. We suggest that this is also attributed to more homogenously distributed particles while conducting the polymerization solvent-free. In the presence of solvents, a phase-separation phenomena might be induced, which results in a lower pore volume of the received carbon material (Table 1). Although chlorine gas is widely used in many industrial processes such as the Kroll process, it should be the attempt of future research to substitute chlorine gas by a green alternative to advance this mechanochemical process to an even more sustainable synthesis.

### Application as supercapacitor electrodes

We selected Carb-SF-3 as electrode material in a symmetrical supercapacitor because of its high specific surface area and pore volume (Table 1). The electrochemical characterization was done in 1 M TEA-BF_4_ in acetonitrile (ACN) and neat EMIM-BF_4_ as an ionic liquid. Since ionic liquids show a lower ion mobility as compared to aqueous or organic electrolytes, a well-connected transport pore system is of particular importance to guarantee a fast ion transport and should result in better power performance [21,56]. Hierarchical pore systems provide enhanced ion transport in meso-/macropores in combination with high energy density due to accessible surface area in micropores [21].

**Figure 7:**
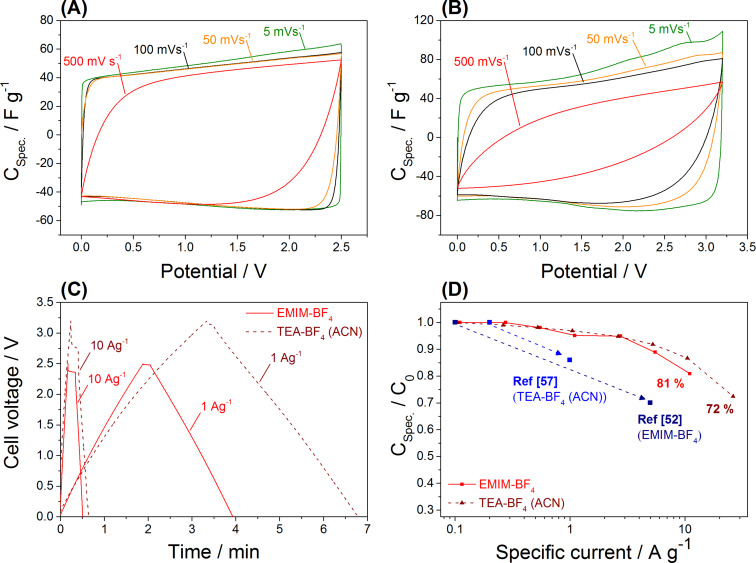
Cyclic voltammograms performed with different scan rates in (A) 1 M TEA-BF_4_ (ACN) and (B) EMIM-BF_4_; galvanostatic charge/discharge curves in two different electrolytes at different specific currents (C); normalized rate capability test with different specific currents (D) in comparison to another Kroll carbon (from [52]) and a mesoporous template carbon (from [57]) with comparable mesopore sizes and surface areas.

The energy storage is accomplished by ion electrosorption as can be inferred from the rectangular shaped cyclic voltammograms (CVs) in both electrolytes (Figure 7A,B) [53]. At low current rates, the material shows good specific capacitance (Table 2) of 138 F g^−1^ in neat EMIM-BF_4_ and 98 F g^−1^ in 1 M TEA-BF_4_ (ACN) determined by galvanostatic cycling with potential limitation at 0.1 A g^−1^. These values are comparable to other known Kroll carbons [52] and non-doped mesoporous carbons [57].

**Table 2:** Electrochemical data summary for sample Carb-SF-3 measured in two different electrolytes.

Sample	Electrolyte^a^	*C*_0_^b^ / F g^−1^	*C*_0_^c^ / F m^−2^	Capacitance loss / %	Specific energy^d^ / Wh kg^−1^	Energy efficiency^e^ / %

Carb-SF-3	O	98	0.054	13^f^	18.99	95.0
Carb-SF-3	IL	138	0.076	19^f^	41.30	83.5
Ref [52]^g^	IL	135	0.072	30^f^	n. d.	n. d.
Ref [57]^h^	O	93	0.062	14^i^	n. d.	n. d.

^a^Electrolyte is abbreviated as followed: O = 1 M TEA-BF_4_, IL = EMIM-BF_4_. ^b^Weigth normalized capacitance at 0.1 A g^−1^. ^c^BET-surface area normalized capacitance at 0.1 A g^−1^, ^d^Specific energy obtained from discharge at 1 A g^−1^ measured in 1 M TEA-BF_4_ (ACN) and at 1 A g^−1^ in EMIM-BF_4_. ^e^Energy efficiency calculated as quotient of the specific energy obtained from discharge and charge at 1 A g^−1^ loss of specific capacitance, calculated as 1 − quotient of *C*_spec_ at 1 A g^−1^ and *C*_0_. ^f^Loss of specific capacitance, calculated as 1 − quotient of *C*_spec_ at 10 A g^−1^ and *C*_0_. ^g^Reference Kroll carbon. ^h^Reference mesoporous carbon. ^i^Loss of specific capacitance, calculated as 1 − quotient of *C*_spec_ at 1 A g^−1^ and *C*_0_ reference non-doped mesoporous carbon.

At a high sweep rate of 500 mV s^−1^, the shape of the CV, recorded in the organic electrolyte (Figure 7A) remains nearly rectangular, which indicated a high power handling ability. The ion mobility of ionic liquids is lower compared to organic electrolytes, as can be seen from the stronger deformation of the CV at high scan rates (Figure 7B). The different rate handling is also quantified by galvanostatic cycling with potential limitation (GCPL) conducted at different specific currents as presented in Figure 7C. At a high current rate of 10 A g^−1^, the specific capacitance was 87% in the organic electrolyte and 81% in EMIM-BF_4_ compared to the specific capacitance at 0.1 A g^−1^. The ability of the carbon to enable a fast charge and discharge is superior compared to other mesoporous non-doped carbon electrodes (Figure 7D). The material also exhibited excellent performance stability, as seen from 92% and 95% after 100 h of floating at 2.7 V for TEA-BF_4_ in ACN and 3.2 V in EMIM-BF_4_, respectively.

## Conclusion

Our work presents a novel, solvent-free approach to receive hierarchical porous carbons with tailorable mesopore volume involving two synthesis steps: firstly, the mechanochemical synthesis of a polymeric composite, received by ball-milling within five minutes only, and secondly, the conversion of this precursor to a hierarchical carbon by a carbochlorination reaction. The received carbons exhibit specific surface areas of up to 1800 m^2^ g^−1^ and high mesopore volumes up to 1.8 cm^3^ g^−1^, making them very attractive for energy applications. When benchmarked as supercapacitor electrode material, Carb-SF-3 shows good specific capacitances with 98 F g^−1^ in 1 M TEA-BF_4_ (ACN) and 138 F g^−1^ in EMIM-BF_4_. Even with high specific currents of 10 A g^−1^ the carbon shows 87% in organic and 91% in ionic liquid electrolyte of its specific capacitance. Moreover, the carbon enables a stable electrochemical performance in both surveyed electrolytes with over 92% capacitance retention after 100 h of voltage floating. Due to the ability to design the mesopore volumes and their relatively narrow pore size distribution, the carbons are also interesting as model carbons for the investigation of different adsorption phenomena.

## Experimental

### Synthesis

Citric acid monohydrate (CA, purity: 95.5%) and titanium isopropoxide (TIPP, purity: 97%) were purchased from Sigma-Aldrich. Ethylene glycol (EG, purity 99.5%) was purchased from Fluka Analytics.

For the solvent-free synthesis of hierarchical porous carbons, 5.25 g CA were ground with 7.10 g TIPP in a molar ratio of 1:1 in a 45 mL ZrO_2_ milling cup for 1 min with 700 rpm. Seven grinding balls out of ZrO_2_ with a diameter of 15 mm were used. Afterwards, different amounts of EG are added and the mixture was ball-milled for another 5 min with 700 rpm. The molar ratio of CA and EG was varied from 1:3 to 1:1. For the liquid-assisted synthesis, 5 mL EtOH were added to the first grinding step.

The resulting polymer was heated to 900 °C at a heating rate of 300 °C h^−1^ in a horizontal tubular furnace under argon atmosphere with a flowrate of 150 mL min^−1^. After 1 h at 900 °C, the gas atmosphere was changed to a mixture of argon (flowrate: 70 mL min^−1^) and chlorine gas (flowrate: 80 mL min^−1^) while the temperature was held for additional 2 h at 900 °C. After cooling to 600 °C under argon, remaining chlorine was removed by hydrogen treatment (flowrate: 80 mL min^−1^) for 1 h.

### Characterization

Nitrogen physisorption experiments were carried out with an AUTOSORB-iQ-C-XR from Quantachrome at −196 °C. Prior to the measurements, the samples were degassed for at least 24 h at 150 °C under vacuum. The specific surface area was calculated in a relative pressure range of 0.05–0.2 per the Brunauer–Emmett–Teller (BET) theory. Values for the total pore volume were determined at a relative pressure of 0.99. Pore size distributions were achieved by applying the hybrid QSDFT model for slit-shaped, cylindrical and spherical pores at −196 °C. The micropore volume was calculated from the cumulative QSDFT pore volume data at 2 nm. Energy dispersive X-ray (EDX) analyses were performed with a SU8020 from Hitachi at an acceleration voltage of 20 kV. Transmission electron microscopy (TEM) was executed with a TEM Libra 200 system from Carl Zeiss Microscopy GmbH with an acceleration voltage of 200 kV. For the TEM, the sample powder was sonicated in acetone for 5 s. A lacey-carbon film on copper net (300 mesh) from Plano was used as TEM grid. Afterward, 5 µL were dropped on the grid and evaporated. IR spectra were conducted with the use of ATR technique, as well as with the DRIFTS technique with a Bruker Vertex 70 in the range of 4000–400 cm^−1^. The hierarchical porous carbon was prepared as free standing electrodes. The carbon material was dispersed in ethanol and we added 10 wt % polytetrafluoroethylene (PTFE, 60 wt % solution in water) as the polymer binder. By crushing the mixture in an agate mortar until the ethanol is evaporated, a dough-like mass was obtained, which was further rolled out until the electrode had a thickness of about 150 µm. The electrode was dried in a vacuum oven at 120 °C for 24 h and we used a disc cutter to obtain electrodes with a diameter of 12 mm. The measurement was done in custom-built cells in a symmetrical two-electrode setup with a quasi-reference electrode out of YP-50F bound with PTFE [58–59]. A 13 mm diameter Whatmann GF/D was used as a separator and 12 mm diameter carbon-coated aluminum discs from MTI Corporation was used as a current collector.

The electrochemical measurements were performed with a Biologic VMP-300 potentiostat/galvanostat. The specific capacitances were calculated with Equation 2 from galvanostatic cycling with potential limitation (GCPL). To compare the electrodes with other materials, they were normalized to their active mass, which is equivalent to the carbon mass in the electrodes, as well as to their specific surface area obtained by the BET method. For the graphical representation of the cyclic voltammograms, the specific capacitances were calculated with Equation 3.

[2]
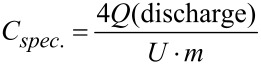


[3]
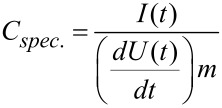


For the calculation of the specific energy of the carbon electrodes in two different electrolytes, Equation 4 was applied with discharge data after the iR drop.

[4]
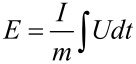


## Supporting Information

File 1 Additional data.
