# Supplementary material for: Mechanochemistry-assisted synthesis of hierarchical porous carbons applied as supercapacitors
Source: Beilstein J Org Chem. 2017 Jul 6;13:1332–41. doi: 10.3762/bjoc.13.130 (PMC5530614; doi:10.3762/bjoc.13.130)
Supplement: File 1 — Additional data. [file Beilstein_J_Org_Chem-13-1332-s001.pdf]

Supporting Information

for

Mechanochemistry-assisted synthesis of hierarchical porous carbons applied as supercapacitors

Desirée Leistenschneider[1], Nicolas Jäckel[2,3], Felix Hippauf[4], Volker Presser[2,3] and Lars Borchardt[1,* §]

Address: [1]Institute of Inorganic Chemistry, Technische Universität Dresden, Dresden, Germany, [2]INM - Leibniz Institute for New Materials & Saarland University, Saarbrücken, Germany, [3]Department of Materials Science and Engineering, Saarland University, Saarbrücken, Germany and [4]Fraunhofer Institute for Material and Beam Technology IWS, Dresden, Germany

*Corresponding author

Email: Lars Borchardt - lars.borchardt@tu-dresden.de,

[§]Tel: +49 35146334960

Additional data

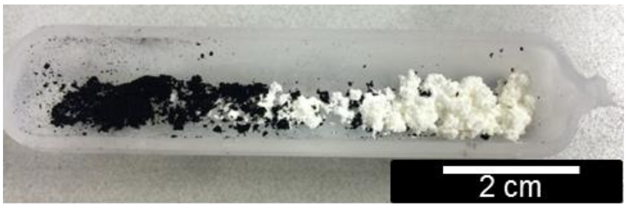

Figure S1: Reference sample Ti citrate after chlorine treatment.

Table S1: Porosity data summary for different carbon samples calculated by QSDFT.

Sample	$SSA_{d<0.7\ nm}$ / $m^2\ g^{-1}$	$SSA_{2\ nm>d>0.7\ nm}$ / $m^2\ g^{-1}$	$SSA_{d>2\ m}$ / $m^2\ g^{-1}$	$V_{d<0.7\ nm}$ / $cm^3\ g^{-1}$	$V_{2\ nm>d>0.7\ nm}$ / $cm^3\ g^{-1}$	$V_{2\ nm>d>0.7\ nm}$ / $cm^3\ g^{-1}$
Carb-SF-1	295	328	804	0.07	0.16	1.11
Carb-SF-2	144	336	1019	0.03	0.16	1.43
Carb-SF-3	181	377	1018	0.04	0.19	1.60
$Carb_{HF}$-LA-3	123	206	438	0.04	0.1	0.48
Carb-LA-3	135	310	1015	0.03	0.15	1.45

Table S2: Composition data of carbon samples determined by elemental analysis.

Sample	C / wt %	H / wt %	N / wt %	S / wt %	Residual mass / wt %
Comp-SF-1	31.2	0.4	1.9	0.0	66.5
Comp-SF-2	35.3	0.4	0.0	0.1	64.2
Comp-SF-3	31.6	0.4	2.2	0.1	65.7
Carb-SF-1	88.9	0.3	0.9	0.7	9.2
Carb-SF-3	92.1	0.4	0.7	0.2	6.6
Carb-LA-3	85.7	0.2	0.0	0.2	13.9

We could not identify the formation of a titanium carbide shell around the embedded TiO_2 particles, preventing any etching by HF by TEM or XRD (Figure S2 and Figure S3).

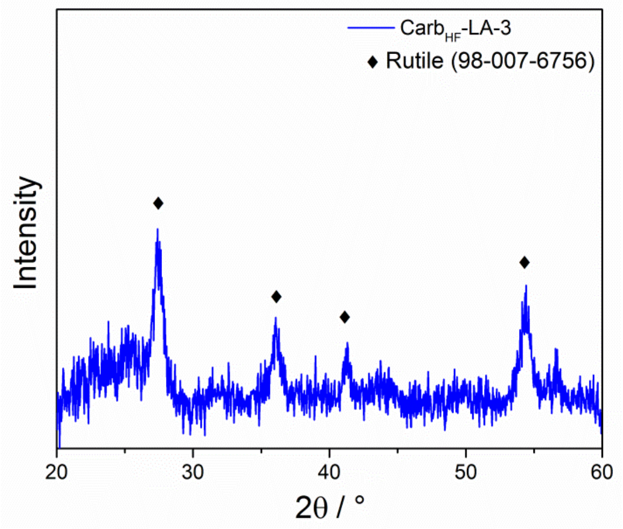

Figure S2: XRD pattern of the sample Carb$_{HF}$-LA-3 with residual rutile and the ideal signal positions for rutile (98-007-6756).

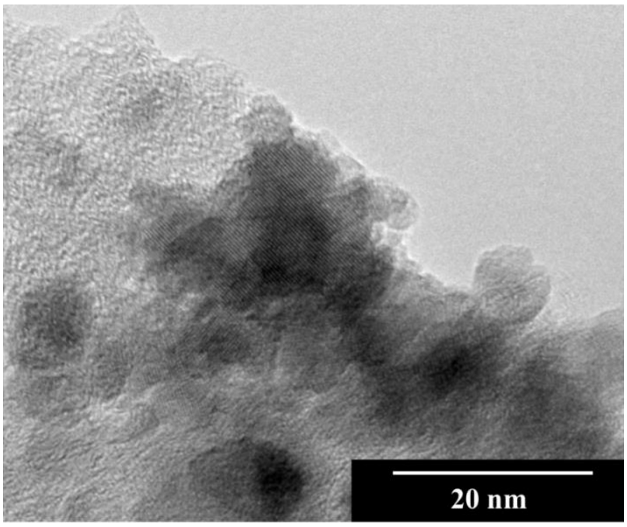

Figure S3: TEM image of sample Comp-SF-3.
